# A case report in cardiovascular magnetic resonance: the contrast agent matters in amyloid

**DOI:** 10.1186/s12880-016-0173-5

**Published:** 2017-01-07

**Authors:** Marianna Fontana, Thomas A. Treibel, Ana Martinez-Naharro, Stefania Rosmini, Raymond Y. Kwong, Julian D. Gillmore, Philip N. Hawkins, James C. Moon

**Affiliations:** 1 National Amyloidosis Centre, University College London, Royal Free Hospital, London, UK; 2 Institute of Cardiovascular Science, University College London, London, UK; 3 Barts Heart Centre, West Smithfield, London, EC1A 7BE UK; 4 Non-Invasive Cardiac Imaging, Cardiovascular Medicine Division, Brigham and Women’s Hospital, Harvard Medical School, Boston, MA USA

**Keywords:** Case report, Amyloidosis, CMR, Contrast

## Abstract

**Background:**

Cardiac amyloidosis is a progressive but underdiagnosed and underappreciated cause of heart failure. In the last few years, cardiovascular magnetic resonance (CMR) has become the gold standard for non invasive diagnosis of cardiac amyloidosis with the characteristic subendocardial late gadolinium enhancement.

**Case presentation:**

We describe a case of a patient who, in the process of aligning protocols for a trial between different centers, had a paired study with two different contrast agents, Dotarem® and MultiHance®. MultiHance® surprisingly failed to demonstrate the characteristic imaging pattern, showing only non specific late gadolinium enhancement at the inferior right ventricular insertion point and different myocardial extracellular volume fraction compared to the one obtained with Dotarem®. MultiHance® is used by many centres, because its partial blood protein binding is a strength for MR angiography, but late gadolinium enhancement, particularly non-ischemic, appears to be compromised.

**Conclusions:**

This case report suggests that contrast agents should be selected with caution, especially with new therapies lining up for amyloid and CMR being used as exploratory end point in clinical trials.

**Electronic supplementary material:**

The online version of this article (doi:10.1186/s12880-016-0173-5) contains supplementary material, which is available to authorized users.

## Background

In the last decade, cardiovascular magnetic resonance (CMR) has grown dramatically and in amyloidosis has become the gold standard for non invasive diagnosis of cardiac involvement with the characteristic subendocardial late gadolinium enhancement (LGE) [1]. The number of new referrals for cardiac amyloidosis has significantly increased in the last few years, with the diagnosis being based on CMR and the characteristic LGE. New contrast agents favoured for their high relaxivity related to blood protein binding, [2] have become available but no one has ever tested the diagnostic performance of these new agents in infiltrative disease.

## Case presentation

**Fig. 1:**
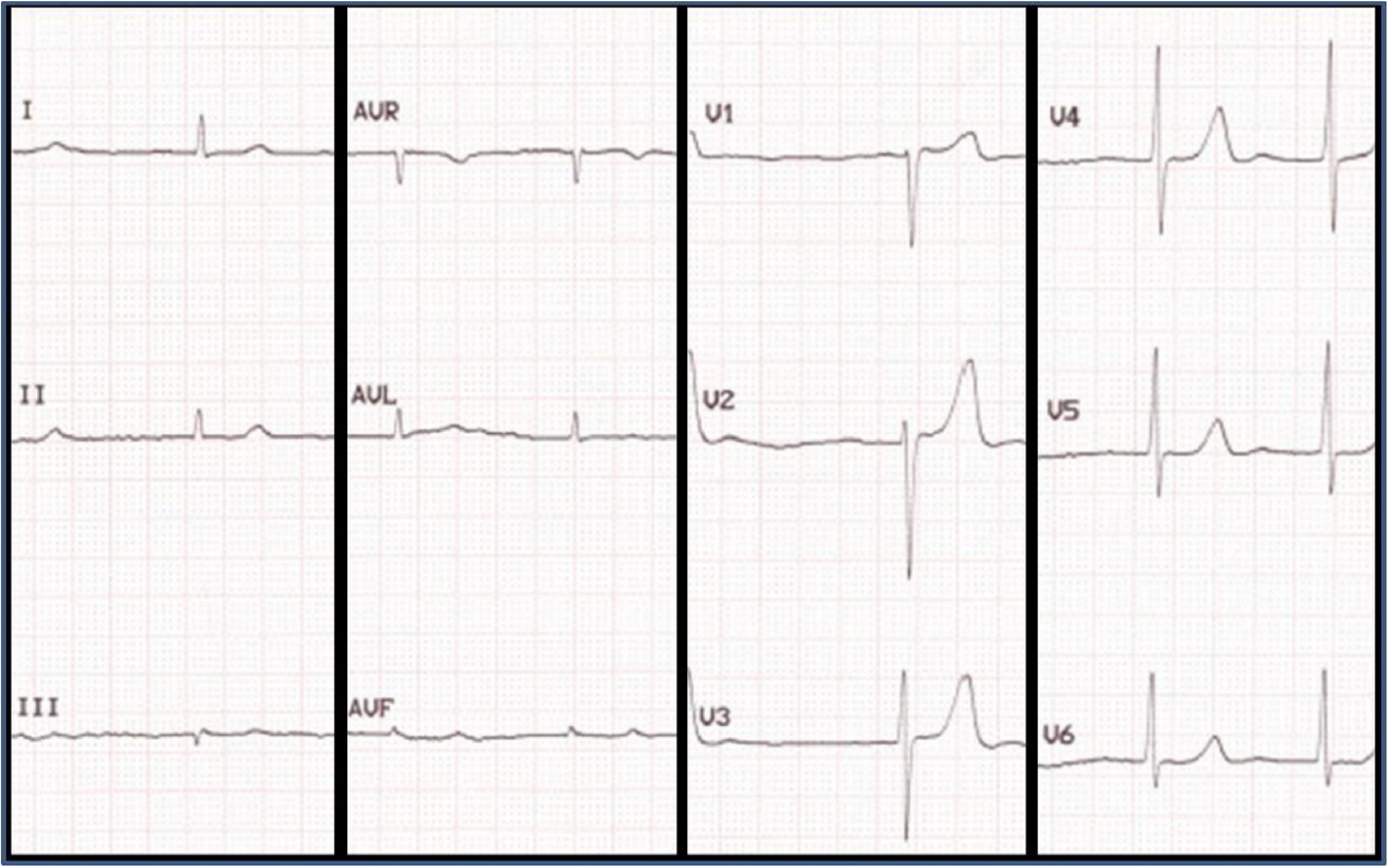
Twelve lead ECG at presentation showing atrial fibrillation

**Fig. 2:**
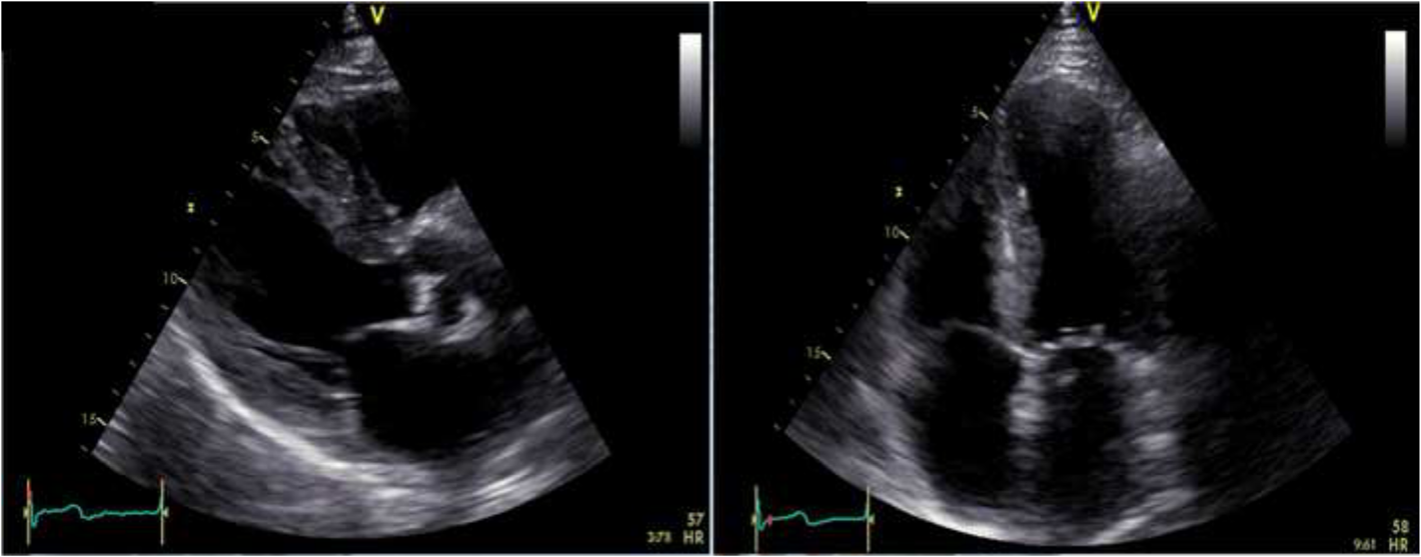
Parasternal long axis (*left*) and apical four-chamber (*right*) views echocardiography images. Videos are available as Additional files 1 and 2

A 77-year old man presented with breathlessness and weight loss. Endoscopy and duodenal biopsy showed amyloid deposits, subtyped as transthyretin type (TTR). Investigations excluded a plasma cell dyscrasia, and showed atrial fibrillation on ECG (Fig. 1), and moderately increased wall thickness with moderate to severe diastolic dysfunction on echocardiography (Fig. 2, videos available as Additional files 1 and 2). TTR gene sequencing showed no mutation so the diagnosis was that of wild-type ATTR amyloidosis (senile systemic amyloidosis). Cardiac involvement, though likely, was not unequivocal as age/hypertension can confound. Technetium-labelled bone scintigraphy and CMR were performed. Scintigraphy demonstrated grade 2 myocardial uptake, highly specific for cardiac amyloidosis. Cine CMR imaging supported the echocardiographic and scintigraphy findings. Tissue characterization showed clear amyloidosis with elevated native myocardial T1 (ShMOLLI, 1072 ms, normal 960 ms+/-30 ms) and, after contrast (Gadoterate meglumine, 0.1 mmol/kg, gadolinium-DOTA, Dotarem® Guerbet S.A. France), abnormal gadolinium kinetics on TI scout (nulling of the myocardium before the blood) and diffuse biventricular subendocardial LGE (Fig. 2) [1]. The extracellular volume fraction (ECV) at 15 min was 0.53.

**Fig. 3:**
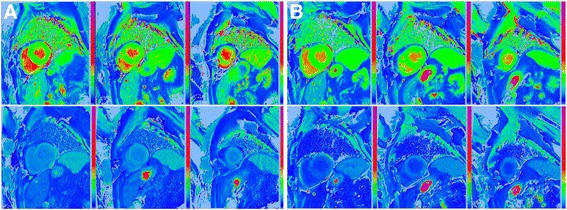
Short axis ShMOLLI T1 mapping images pre-contrast (top) and post-contrast (bottom) with gadolinium-DOTA, Dotarem® (**a**) and with gadolinium-BOPTA, MultiHance® (**b**). Myocardial T1 post-contrast = 486 ms and blood T1 post-contrast =506 ms 15 min after gadolinium-DOTA, Dotarem®; and myocardial T1 post-contrast = 344 ms and blood T1 post-contrast = 287 ms 15 min after gadolinium-BOPTA, MultiHance®

**Fig. 4:**
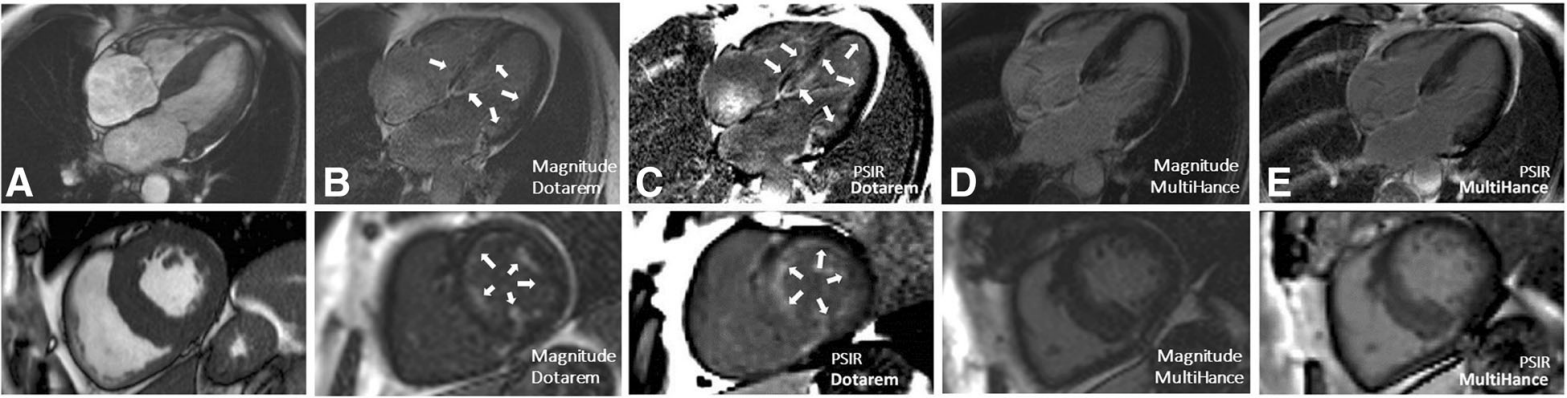
CMR end-diastolic cine (**a**); late gadolinium images with Magnitude-FLASH (**b**) and late gadolinium images with PSIR-FLASH (**c**) after gadolinium-DOTA, Dotarem® showing the classic amyloid global, subendocardial late gadolinium enhancement pattern; late gadolinium images with Magnitude-FLASH (**d**) and late gadolinium images with PSIR-FLASH (**e**) after gadolinium-BOPTA MultiHance®, showing no features of amyloid

Two weeks later, the CMR was repeated (same scanner and team with no changes in the patient regarding treatment or disease status) within the screening tests of a clinical trial, this time with a different contrast agent, gadobenate dimeglumine (0.1 mmol/kg, gadolinium-BOPTA, MultiHance®, Bracco, Milan, Italy). The cines and native T1 findings were the same (Fig. 3). The LGE however was markedly different – the subendocardial LGE so characteristic of amyloidosis was not visible, even with phase-sensitive inversion recovery (PSIR). There was just non-specific right ventricle insertion point LGE (Fig. 4). The ECV at 15 min obtained using Gd-BOPTA (MultiHance®) was now lower – 0.45 compared to 0.53 with gadolinium-DOTA (Dotarem®) (same acquisition time after contrast administration). This difference is higher than the expected variability of the technique. Prior publications demonstrate the ECV to be stable to a standard deviation of 0.02 (0.03 for amyloid); [3] here a 0.08 difference with contrast agent is recorded.

## Discussion

Gd-BOPTA (MultiHance®) is an ionic linear chelate favoured for its high relaxivity related to blood protein binding, [2] which also makes it a partially “intra-vascular” contrast agent. Here, paired scanning performed serendipitously for a clinical trial failed to demonstrate the characteristic pattern of myocardial amyloidosis, and performed poorly compared to gadoterate meglumine. MultiHance® is widely used for CMR. This effect was unexpected – but theoretical concerns had been raised for ECV mapping with protein bound contrast agents [4]. Due to difference in relaxivity between the contrast agents, some Authors proposed the use of a lower dose of Gd-BOPTA for reaching similar T1 and ECV values. In this case the same dose was used for both contrasts and this could explain in part the differences observed. In this case, the use of MultiHance® did not affect the diagnosis, but reporting the MultiHance® CMR in isolation without the previous work-up by the National Amyloid Centre could have conceivably led to a misdiagnosis.

## Conclusion

We suggest that protein bound agents for interstitial enhancement of non-ischaemic cardiomyopathy (myocardium, ECV measurement) should be used with caution whilst further work is undertaken as the diagnostic performance may not be the same as non-protein bound variants.

## Additional files

Additional file 1: Video 1. Echocardiogram: apical four chamber view. (AVI 5448 kb)
Additional file 2: Video 2. Echocardiogram: parasternal long axis view. (AVI 5765 kb)

## Abbreviations

CMR: Cardiovascular magnetic resonance
ECG: Electrocardiogram
ECV: Extracellular volume
Gd: Gadolinium
LGE: Late gadolinium enhancement
PSIR: Phase Sensitive Inversion Recovery
ShMOLLI: Shortened Modified Look-Locker Inversion recovery
TTR: Transthyretin type
